# Concordance of PD-L1 Status Between Image-Guided Percutaneous Biopsies and Matched Surgical Specimen in Non-Small Cell Lung Cancer

**DOI:** 10.3389/fonc.2020.551367

**Published:** 2021-02-23

**Authors:** Liang Zhao, Peiqiong Chen, Kaili Fu, Jinluan Li, Yaqing Dai, Yuhuan Wang, Yanzhen Zhuang, Long Sun, Haojun Chen, Qin Lin

**Affiliations:** ^1^ Department of Radiation Oncology, The First Affiliated Hospital of Xiamen University, Teaching Hospital of Fujian Medical University, Xiamen, China; ^2^ Department of Pathology, The First Affiliated Hospital of Xiamen University, Teaching Hospital of Fujian Medical University, Xiamen, China; ^3^ Department of Nuclear Medicine & Minnan PET Center, The First Affiliated Hospital of Xiamen University, Teaching Hospital of Fujian Medical University, Xiamen, China

**Keywords:** programmed death-ligand 1 (PD-L1), biopsy, surgical resected specimen, PET/CT, non-small cell lung cancer

## Abstract

**Objective:**

Programmed death-ligand 1 (PD-L1) expression status is a crucial index for identifying patients who will benefit from anti-programmed cell death protein 1 (PD-1)/PD-L1 therapy for non-small cell lung cancer (NSCLC). However, the concordance of Tumor Proportion Score (TPS) between biopsies and matched surgical specimens remains controversial. This study aims to evaluate the concordance of PD-L1 expression between image-guided percutaneous biopsies and matched surgical specimens.

**Method:**

We evaluated 157 patients diagnosed with operable NSCLC on both surgical tissue sections and matched lung biopsies retrospectively. The patients underwent either regular computed tomography (CT)-guided biopsy (n = 82) or positron emission tomography (PET)/CT-guided biopsy (n = 75). The concordance between surgical specimens and lung biopsies for PD-L1 TPS was evaluated using Cohen’s kappa (κ) coefficient.

**Results:**

Immunohistochemical expression of PD-L1 was evaluated in both surgical resected specimens and matched biopsies in the eligible 138 patients. The concordance rate of PD-L1 expression between surgical tissue sections and matched biopsies was fairly high at 84.1% (116/138), and the κ value was 0.73 (95% CI: 0.63–0.83, P < 0.001). The concordance rate was higher for tissue sections from PET/CT-guided biopsy than for tissue sections from CT-guided biopsy [88.6% (62/70, κ value: 0.81) vs 79.4% (54/68, κ value: 0.66)].

**Conclusion:**

PD-L1 TPS was strongly concordant between surgical specimens and matched lung biopsies. Thus, the routine evaluation of PD-L1 expression in diagnostic percutaneous biopsies could be reliable for identifying patients who will benefit from anti-PD-1/PD-L1 immunotherapy.

## Introduction

Lung cancer is the leading cause of cancer-related death worldwide, and it is projected to account for over 22% of deaths in 2020 in the United States (1). Non-small cell lung cancer (NSCLC) accounts for about 80% of lung cancers. NSCLC has poor prognosis, but immunotherapy has markedly improved survival in patients with advanced NSCLC without driver alterations. In the KEYNOTE-024 and KEYNOTE-042 phase III clinical trials, programmed cell death protein 1 (PD-1)-targeted immunotherapy significantly improved the overall survival (OS) rate compared with standard therapy in advanced NSCLC (2, 3). Based on these studies, the FDA approved pembrolizumab as the first-line treatment for advanced NSCLC patients with a Tumor Proportion Score (TPS) of ≥50% (based on KEYNOTE-024) and 1% (based on KEYNOTE-042), respectively.

Other immunotherapies targeted against PD-1 or programmed death-ligand 1 (PD-L1), such as nivolumab, atezolizumab, and durvalumab (**Supplemental Table 1**), also showed OS benefit with similar PD-L1 cut-off values (4, 5). Thus, determining PD-L1 expression status may help select patients who will optimally benefit from immunotherapy. Immunohistochemistry (IHC) has been widely used to evaluate PD-L1 status in both clinical trials and routine clinical practice. Currently, PD-L1 testing is mainly performed on biopsy samples, which may not be representative of the whole tumor. Thus, the concordance of TPS between lung biopsies and matched surgical specimens remains controversial (6), which may result in decreased confidence on the reliability of biopsies for PD-L1 testing.

Image-guided percutaneous biopsy is currently widely used as it is minimally invasive and is associated with fewer complications. CT-guided biopsy is the most common approach for sampling of lung lesions, but it has varying diagnostic performance depending upon the target organ and type of needle used (7, 8). Meanwhile, ^18^F-FDG PET/CT provides both anatomic structures and metabolic features and has therefore been suggested to improve the diagnostic accuracy of image-guided biopsy (9–12). We have previously reported the importance of PET/CT-guided biopsy for patient evaluation at various stages of cancers (9, 13, 14). Applying PET/CT information to image-guided biopsy may facilitate accurate histopathological diagnosis and help with staging. However, only few studies to date have compared the reliability and reproducibility of PET/CT-guided biopsy with corresponding resected surgical specimens. Moreover, the use of PD-L1 as a biomarker for determining sensitivity to PD-1/PD-L1 checkpoint blockades has raised concerns on the reliability of biopsy samples compared with surgical specimens. Thus, the present study aimed to evaluate the concordance of immunohistochemical expression of PD-L1 between image-guided biopsies and matched surgical specimens.

## Materials and Methods

### Patient Population

The study protocol was discussed and approved by the institutional review board of the First Affiliated Hospital of Xiamen University (ID, KY2017-001), and written informed consent was obtained from all patients.

We retrospectively evaluated 157 patients with operable NSCLC who underwent both diagnostic percutaneous lung biopsy and surgical resection at the First Affiliated Hospital of Xiamen University, between December 2016 and October 2019. The exclusion criteria were as follows: (i) history of chemotherapy or radiotherapy before biopsy and surgery and (ii) incomplete preoperative clinical record, including data on body mass index, smoking history, blood routine examination, and blood biochemistry examination. Of the 157 patients, 82 underwent CT-guided biopsy, while the other 75 patients underwent ^18^F-FDG PET/CT-guided biopsy. Eventually, 138 matched biopsy and surgical resection specimens were obtained for further IHC analysis of PD-L1 expression. There were 72 men and 66 women in the derivation cohort, and the median patient age was 63 years (range: 25–81 years). In total, 53 (38.4%) patients were current or former smokers. All patients were staged or restaged according to the 8th International Association for the Study of Lung Cancer staging system based on postoperative pathological result. Overall, 61 (44.2%) patients had stages II–III disease, and majority (n = 116, 84.1%) had non-squamous histology. The clinicodemographic characteristics of the 138 patients are summarized in **Table 1**.

**Table 1:** Clinicodemographic characteristics of the study patients.

Characteristic	Number	%
Age (years)		
<65	79	57.2%
≥65	59	42.8%
Median (range)	63 (25–81)	
Sex		
Male	72	52.2%
Female	66	47.8%
Smoking history		
Nonsmoker	85	61.6%
Smoker	53	38.4%
Histology		
Squamous cell carcinoma	22	15.9%
Adenocarcinoma	116	84.1%
Clinical stage (International Association for the Study of Lung Cancer 8th)		
I	77	55.8%
II/III	61	44.2%
Tumor size (cm)		
≤3	94	68.1%
>3	44	31.9%
Derived neutrophil–lymphocyte ratio		
<3	133	96.4%
≥3	5	3.6%
Lactate dehydrogenase (U/L)		
<240	129	93.5%
≥240	9	6.5%
Body mass index		
<25	102	73.9%
≥25	36	26.1%

### Image-Guided Percutaneous Biopsy

Diagnostic percutaneous lung biopsy was performed under either CT guidance or ^18^F-FDG PET/CT guidance. ^18^F-FDG PET/CT protocol and imaging analysis were performed as previously described (15). The imaging modality was determined based on discussions with the referring oncologists. The biopsy target was decided by the referring oncologists and interventional radiologists based on the results of ^18^F-FDG PET/CT or CT scan.

Image-guided biopsy was performed with an 18G or 20G semiautomatic core needle (Coaxial Achieve, Bard, IL, USA) with coaxial guide needle. The percutaneous biopsy was performed by a board-certified interventional radiologist following a step-by-step technique as previously described (8, 13). Briefly, the patients were positioned in a supine or prone position in accordance with factors such as the location of FDG-avid lesion, shortest skin-to-target distance, and optimal needle path. Interventions were conducted under aseptic conditions after administration of local anesthesia using 2.0% lidocaine. The needle was introduced in a stepwise manner under fused PET/CT and CT imaging guidance. Three or four specimens were obtained from each patient, after which histopathological examination and immunohistochemical staining were performed on each specimen. After the biopsy, the patients were observed by referring oncologists for at least 24 h, and the patients were asked to report any abnormality.

### Immunohistochemistry

The samples were prepared and stained as previously described (15). In brief, formalin-fixed, paraffin-embedded tumor tissues were sliced into 4 μm-thick sections. For IHC detection of PD-L1, we used the BenchMark GX automated slide stainer (SP263, Ventana, Oro Valley, AZ, USA) to stain the sections with the PD-L1 antibody according to the manufacturer’s recommended protocol. Positive control (placenta) and negative control samples were run simultaneously.

The immunostained tissue sections were scored according to the PD-L1 scoring algorithm (16, 17) by three independent experienced pathologists who were blinded to the clinical data. Discrepancies in the PD-L1 score were resolved by reviewing the slides again. The cut-off values for PD-L1 expression were set to 1 and 50%.

### Statistical Analysis

All statistical analyses were conducted using the SPSS 22.0 statistical analysis software (IBM, Armonk, NY, USA). For continuous data, we used the t-test or the Wilcoxon test for analyses, as appropriate. The concordance of PD-L1 TPS between surgical specimens and lung biopsies was evaluated using Cohen’s kappa (κ) coefficient (18, 19). The relative strength of agreement was interpreted as follows: κ <0, poor; 0.01–0.20, slight; 0.21–0.40, fair; 0.41–0.60, moderate; 0.61–0.80, substantial; and 0.81–1.00, almost perfect (18, 19). Between group comparisons were performed using the chi-squared test, Yates’ correction of chi-squared test, or Fisher’s exact test. The correlation between different variables was analyzed using the non-parametric Spearman’s rank test. All tests were two-sided, and a *P* value lower than 0.05 was considered statistically significant.

## Results

**Figure 1:**
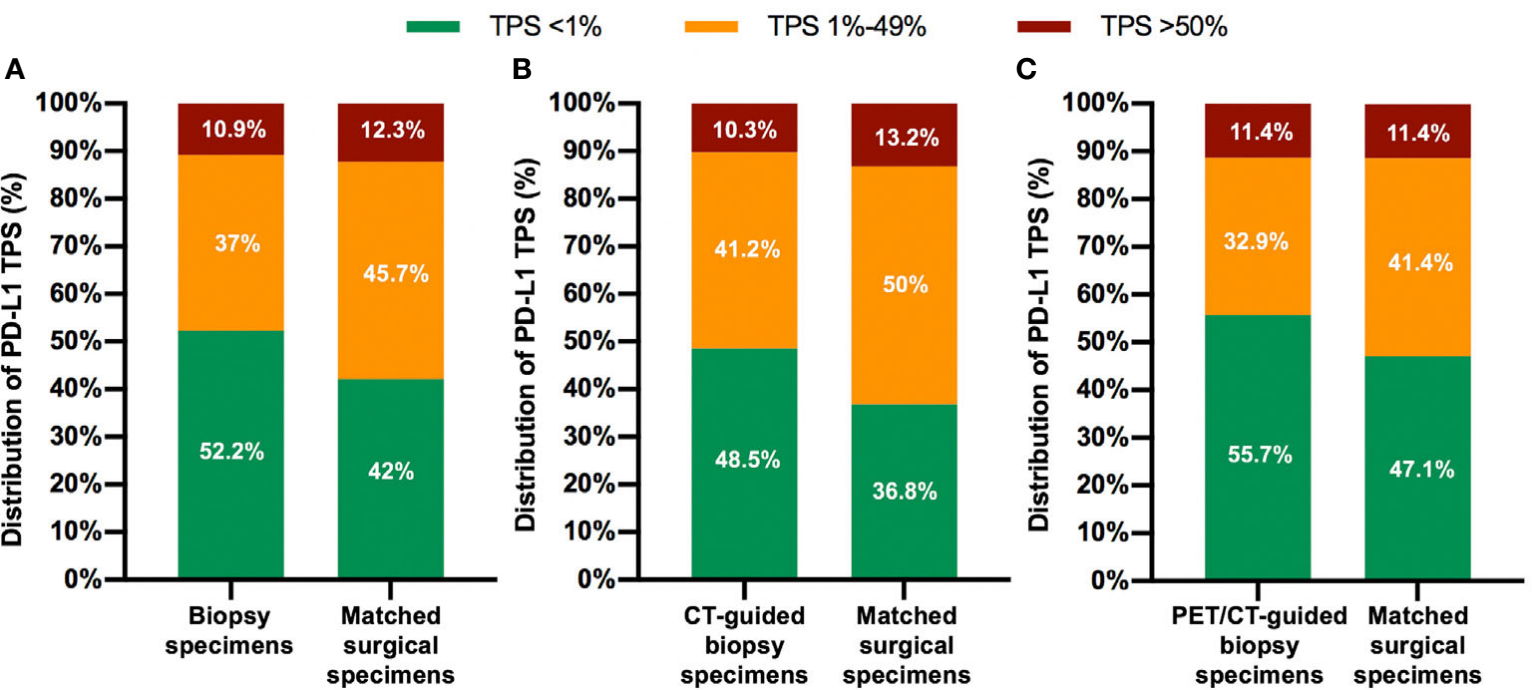
Distribution of PD-L1 Tumor Proportion Score (TPS) for both biopsy specimens and matched surgical specimens in the overall patient population **(A)**, in the CT-guided biopsy group **(B)**, and in the PET/CT-guided biopsy group **(C)**.

The diagnostic success rates of CT- and PET/CT-guided biopsy were 82.9% (68/82) and 93.3% (70/75), respectively. There were 11 central and 146 peripheral NSCLCs. Fourteen (8.9%) patients underwent pneumothorax, while 10 (6.4%) patients happened hemoptysis. PD-L1 TPS was <1, 1–49, and ≥50% in 72, 51, and 15 percutaneous biopsy specimens, respectively, and in 58, 63, and 17 surgical resection specimens, respectively (**Figure 1A**). Compared with the whole tumor section, 19 biopsy specimens underestimated the PD-L1 TPS, while only three biopsy section overestimated the TPS. Regarding the concern that biopsy specimens may underestimate the PD-L1 expression, four cases (one with PET/CT-guided biopsy, and three with CT-guided biopsy) in our study showed lower PD-L1 TPS in diagnostic biopsies (1–49%) as compared to the surgical samples (> 50%). The tissue sections from the biopsy and surgical resection in the four cases were further cautiously analyzed by our experienced pathologists, and a highly heterogenous expression of PD-L1 in the surgical specimen was observed in two out of four cases. There was no significant difference in PD-L1 TPS between biopsy and surgical tissue sections (P = 0.24).

**Figure 2:**
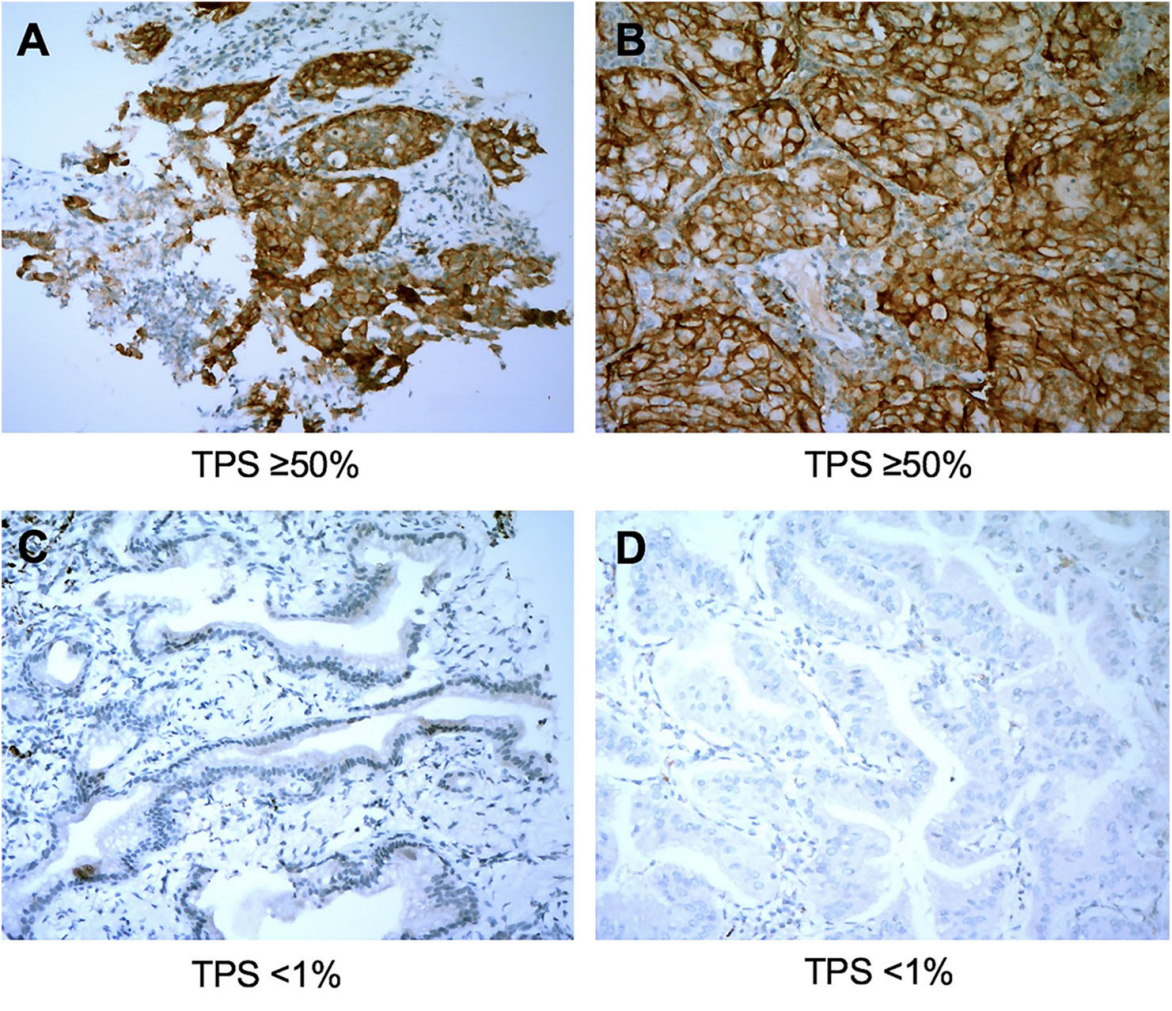
Representative images of concordant cases between biopsy specimens (left panel) and matched surgical resection specimens (right panel). The PD-L1 Tumor Proportion Score (TPS) in the biopsy tumor specimen **(A)** and the corresponding resected tumor **(B)** were both ≥50%. PD-L1 TPS in the biopsy tumor **(C)** and the matched resected specimen **(D)** were both <1%. All images are at ×40 magnification.

**Figure 3:**
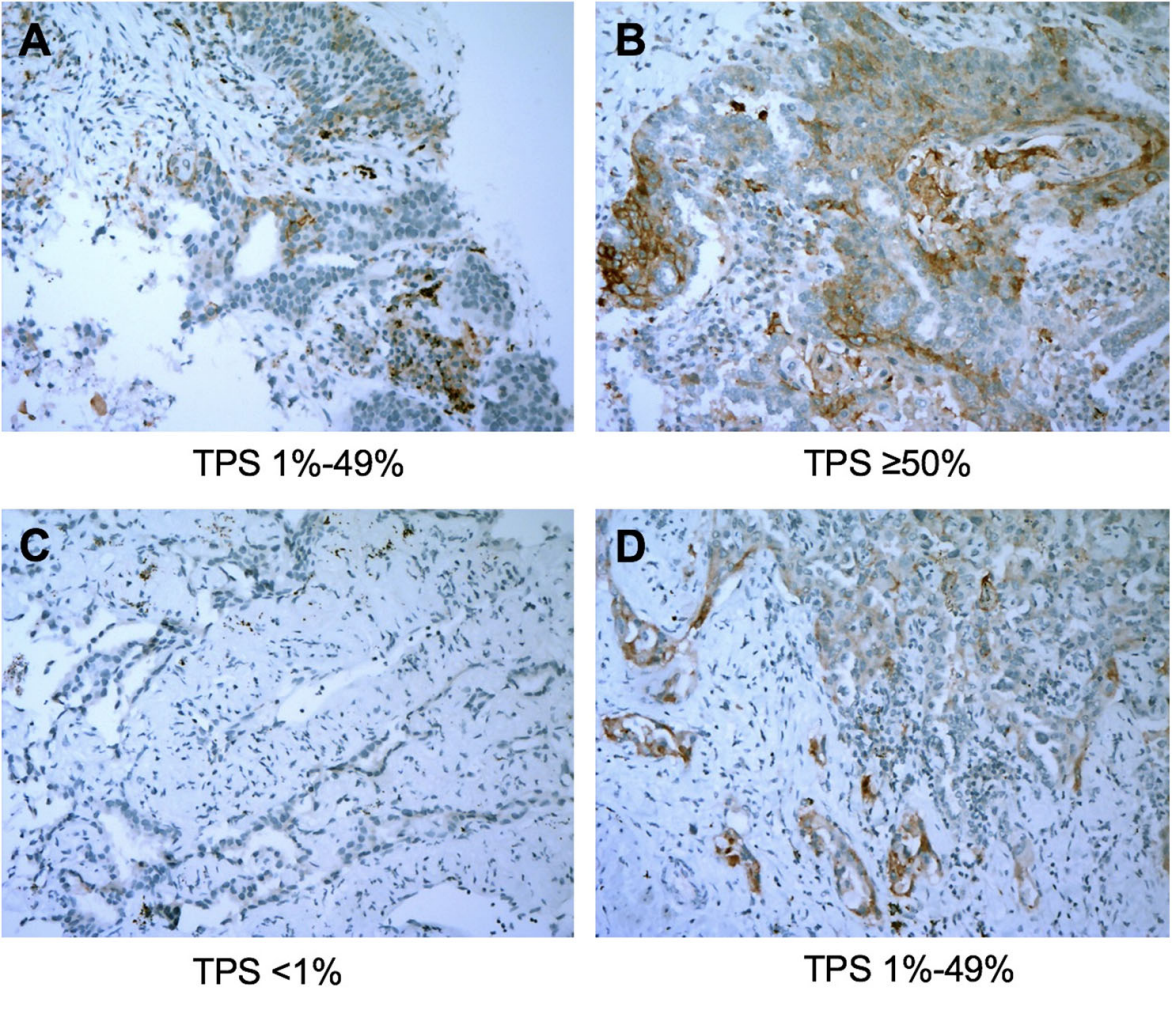
Representative images of discordant cases between biopsy specimens (left panel) and matched resected specimens (right panel). The PD-L1 Tumor Proportion Score (TPS) was 10% in the biopsy tumor specimen **(A)** and was ≥50% in the corresponding resected tumor **(B)**. PD-L1 TPS in the biopsy specimen was <1% **(C)** and was 20% in the matched surgical specimen **(D)**. All images are at ×40 magnification.

The overall concordance rate for PD-L1 TPS between percutaneous biopsy specimens and matched surgical specimens was 84.1% (116/138). The Cohen’s κ value was equal to 0.73 (95% CI: 0.63–0.83, P < 0.001), indicating substantial agreement. The concordance rate at a cut-off value of 1% PD-L1 TPS was 88.4%, and the κ value was equal to 0.77. The concordance rate at a cut-off value of PD-L1 TPS 50% also indicated substantial agreement (κ = 0.79). Representative images of concordant and discordant PD-L1 between biopsy and matched surgical specimens are shown in **Figures 2** and **3**, respectively. The Cohen’s κ value according to histological subtypes was 0.79 (95% CI: 0.57–1, P < 0.001) for squamous cell carcinoma and 0.71 (95% CI: 0.59–0.83, P < 0.001) for lung adenocarcinoma.

**Figure 4:**
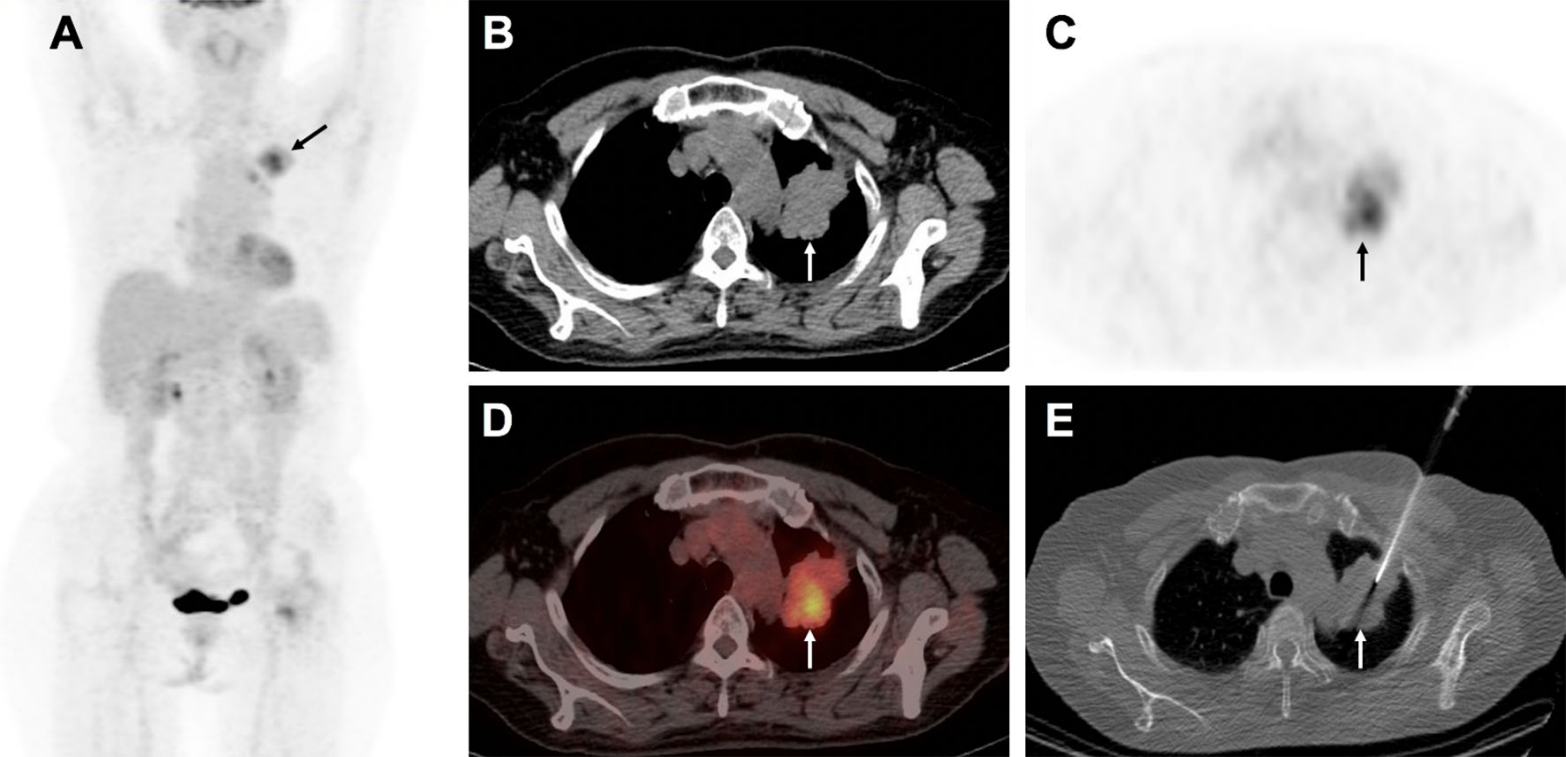
A 58-year-old man with suspected lung carcinoma underwent ^18^F-FDG PET/CT for tumor staging before treatment. The maximum intensity projection image **(A)** shows intense activity in the left upper lobe of the lung (arrow). Axial view of PET/CT **(B)** CT scan, **(C)** PET image, **(D)**, combined PET/CT image, shows the metabolic active mass (arrow) with atelectasis in its caudal part. Axial view of CT image **(E)** shows biopsy needle positioned into the metabolic active area (arrow). Histological examination confirmed the lung lesion as primary adenocarcinoma.

There was no statistical difference in the distribution of PD-L1 TPS between biopsy and surgical specimens (**Figures 1B, C**). The concordance rate for PD-L1 TPS between CT-guided biopsy and matched surgical tissue was 79.4% (54/68), and the Cohen’s κ value was 0.66 (95% CI: 0.50–0.82, P < 0.001), which indicated substantial agreement. Meanwhile, the concordance rate between PET/CT-guided biopsy specimen and matched surgical resection specimen was higher at 88.6% (62/70), and the Cohen’s κ value was 0.81 (95% CI: 0.68–0.94, P < 0.001), which indicated almost perfect agreement (**Table 2**). PD-L1 TPS was significantly associated with SUVmax on Spearman correction analysis (P = 0.048, **Supplemental Table 2**). Representative ^18^F-FDG PET/CT images for lung biopsy are shown in **Figure 4**.

**Table 2:** Cohen’s κ for concordance between percutaneous biopsy and matched surgical specimens based on TPS.

PD-L1 cutoff	Cohen’s kappa (κ)		
	Overall population (N = 138)	CT group (N = 68)	PET/CT group (N = 70)
**Tumor Proportion Score (TPS) ≥1%**	0.77	0.7	0.83
**TPS ≥50%**	0.79	0.72	0.86
**Overall**	0.73	0.66	0.81

As the CT and PET/CT groups had different concordance between percutaneous biopsy and matched surgical specimens, between-group comparisons were performed. The results showed no signifcant difference in the measured PD-L1 TPS between CT-guided biopsies and PET/CT-guided biopsies (P = 0.47). There was no significant difference in the primary clinicodemographic characteristics between the two cohorts (**Supplemental Table 3**).

## Discussion

Anti-PD-1/PD-L1 immunotherapy has improved the prognosis of NSCLC. In particular, nivolumab and pembrolizumab have significantly improved the long-term survival of patients with NSCLC (20, 21). Compared to the usual OS rate of 5% with standard chemotherapy, the 5-year OS rate of pembrolizumab treatment was 29.6% in NSCLC patients with a PD-L1 TPS of over 50% in the KEYNOTE-001 study (21). Therefore, determining the PD-L1 TPS may help identify patients who will benefit from PD-1/PD-L1 blockade therapy, allowing for a more individualized treatment approach and avoiding unnecessary treatment. In this study, the percentage of NSCLCs with PD-L1 TPS <1, 1–49, and ≥50% were 42.0, 45.7, and 12.3%, respectively, which was in agreement with the previous publication (22). Accordingly, our study evaluated the concordance of tumor PD-L1 expression between image-guided percutaneous biopsies and matched surgical specimens in patients with NSCLC. Our results indicated substantial agreement of the PD-L1 TPS between surgical specimens and matched lung biopsies. Notably, samples from PET/CT-guided biopsy demonstrated higher success rate and concordance with surgical tissue sections than those from CT-guided biopsy.

PD-L1 testing using surgical specimens is rarely feasible in patients with NSCLC because of diagnosis at the advanced stage. Our results support the reliability of PD-L1 TPS determined using image-guided percutaneous biopsy specimens. These results can help establish PD-L1 expression assessed using image-guided biopsy specimen as a reliable biomarker for predicting benefit from anti-PD-1/PD-L1 immunotherapy.

Previous investigations have shown that intratumoral heterogeneity of PD-L1 expression exists within the entire surgical specimen (23), which may result in low concordance with the results obtained on diagnostic biopsies. As such, the concordance of PD-L1 status between biopsy samples and matched resected specimens varied across previous studies. For example, Tsunoda et al. and Kitazono et al. showed good concordance (24, 25), whereas Ilie et al. and Erik et al. demonstrated poor concordance (6, 26). It is worth noting that the biopsy samples in all of these studies were mainly obtained from bronchoscopic biopsy (includes EBUS-TBNA, transbronchial or endobronchial biopsy), and part of the specimen were metastatic lymph nodes instead of primary lung tumors (6, 24–26). Despite being minimally invasive, a tissue core usually is not obtained with bronchoscopic biopsy, limiting a detailed morphologic examination and therefore (27), may affect the observation on PD-L1 expression of tumor cell membrane. In our study, all biopsy specimens were obtained percutaneously using image guidance, which allowed sufficient tissues (generally 3–4 biopsy fragments were obtained for each patient, 8–12 mm * 0.5 mm for each fragment) than bronchoscopic biopsy and tissue microarrays. Although previous study reported a significant difference regarding the number of biopsy tissues between concordant and discordant case (6), we still cannot conclude that more sufficient tissues obtained *via* percutaneous biopsy is the reason for the high PD-L1 concordance observed in our study relative to studies wherein most tissue was obtained with bronchoscopy. Further investigation that comparing percutaneous to bronchoscopic biopsy with respect to PD-L1 testing, is in of itself an important subject matter, but not one addressed in this study.

A successful biopsy procedure must provide a diagnostic sample of tissue, which means the sample of tissue must adequately show the presence of malignancy and specific histopathologic features. Although, PET/CT-guided percutaneously obtained biopsy is not the universal standard of care for image guided biopsies, PET/CT guidance has been widely used to improve the biopsy success rate (9–12). In our study, PET/CT-guided biopsy samples demonstrated a higher success rate (93.3%) than did CT-guided biopsy samples (82.9%), and no significant difference regarding patients’ characteristics was observed between those who underwent PET/CT-guided biopsy and CT-guided biopsy. According to our experience, PET/CT-guided biopsy demonstrates the following advantages: First, reducing the frequency of sampling failure can improve biopsy results and patient experiences. Second, resulting in a higher percentage of malignant lesions than did CT-guided biopsy, which could be explained by better identification of the lesion site based on the integration of anatomic structure with metabolic features. For example, we found that ^18^F-FDG PET/CT-guided biopsy has better accuracy in NSCLC with pulmonary atelectasis in this study (**Figure 4**). Moreover, PET/CT allows identification of FDG-avid lesions that are most accessible to biopsy from among multiple lesions with similar uptake (9–11). Biopsy of the most accessible lesion could simultaneously reduce the risk of complications and minimize the sampling error.

Another important finding of this study is that samples from PET/CT-guided biopsy showed stronger concordance with surgical tissue sections than those from CT-guided biopsy, despite that, there was no significant between-group difference in patient characteristics. Considering the possibility of intra-tumor heterogeneity of PD-L1 expression in NSCLC, sampling the specimen that accurately reflects the PD-L1 status is important (28). Increasing evidence show ^18^F-FDG uptake in NSCLC samples was positively correlated with PD-L1 expression (29–32), which has been further confirmed in our study. This might be one of the reasons that the FDG uptake of the primary lesion was able to predict the immunotherapy response (33, 34). In some cases, ^18^F-FDG PET/CT can show the heterogeneity of metabolism in lesions, which are of equal density in CT. Puncture sampling of areas with ^18^F-FDG-avid focus may help obtain representative specimens for measuring PD-L1 expression.

Compared with previous investigations, our study has the following advantages. First, we compared PD-L1 expression between surgical tissue sections and image-guided percutaneous biopsies in the same group of patients. In the ATLANTIC study that included over 1500 NSCLC patients, PD-L1 expression status was not significantly different between biopsy and surgical samples (35). However, the samples (1,365 samples were obtained by biopsy and 180 by surgical resection) were not matched from the same patients. Second, we used relatively new samples for PD-L1 staining; the specimen in our cohort was less than 3 years old. A previous study showed that samples older than 3 years may show lower PD-L1 TPS on IHC (35). Third, we used SP263 as the antibody for PD-L1 measurement, and the cut-off value was clinically relevant (2–5). SP263 antibody in PD-L1 assay has been reported to have high reliability and reproducibility for NSCLC tumor samples (16, 36, 37). Blueprint PD-L1 IHC Assay Comparability Project Phase 2 consolidates the analytical evidence for interchangeability of the 22C3, SP263, and 28-8 assays because of the similar analytical performance (38).

Despite its advantages, our study also has some notable limitations that need to be addressed. First, our cohort did not include patients with stage IV NSCLC, and all patients had resectable disease. Consequently, the concordant between biopsy samples and matched surgical specimens was mostly applied to early stage NSCLCs, rather than advanced disease. Second, anti-PD-1/PD-L1 immunotherapy to date was mainly used in unresectable NSCLC patients, but the patients in our cohort were treated with surgery rather than immunotherapy. However, the apparent survival benefit of immunotherapy has transformed it from being an alternative modality to being the recommended first-line treatment in the real world. Anti-PD-1/PD-L1 immunotherapy in the neoadjuvant setting also showed encouraging results in patients with resectable lung cancer (39, 40). Third, we did not examine multiple areas of the surgical specimens to evaluate the PD-L1 expression. Since the PD-L1 protein levels in NSCLC reveals heterogeneity within tumors, the PD-L1 expression of the whole tumor in this study may not be fully evaluated. Additionally, this was a retrospective, single-center study with a moderate number of patients. Prospective, multicenter studies with a larger patient population are needed.

In conclusion, PD-L1 expression is concordant between diagnostic percutaneous biopsy samples and matched surgical specimens. Thus, PD-L1 expression in image-guided percutaneous biopsies could be a reliable biomarker for screening patients who will benefit from anti-PD-1/PD-L1 immunotherapy.

## Data Availability Statement

The raw data supporting the conclusions of this article will be made available by the authors, without undue reservation.

## Ethics Statement

The studies involving human participants were reviewed and approved by Clinical Research Ethics Committee of the First Affiliated Hospital of Xiamen University. The patients/participants provided their written informed consent to participate in this study.

## Author Contributions

LZ, HC, and QL: study conception and design. KF and YD: literature research. KF, PC, YW, and YZ: data acquisition. PC, JL, YD, and LS: data analysis and interpretation. LZ and HC: manuscript drafting. HC and LZ: manuscript editing. All authors contributed to the article and approved the submitted version.

## Funding

This work was supported by the National Natural Science Foundation of China (Grant number 81772893 and 81701736).

## Conflict of Interest

The authors declare that the research was conducted in the absence of any commercial or financial relationships that could be construed as a potential conflict of interest.

## References

[1] Siegel RL Miller KD Jemal A. Cancer statistics, 2020. CA Cancer J Clin (2020) 70(1):7–30. 10.3322/caac.21590 31912902

[2] Reck M Rodriguez-Abreu D Robinson AG Hui R Csoszi T Fulop A. Pembrolizumab versus Chemotherapy for PD-L1-Positive Non-Small-Cell Lung Cancer. N Engl J Med (2016) 375(19):1823–33. 10.1056/NEJMoa1606774 27718847

[3] Mok TSK Wu YL Kudaba I Kowalski DM Cho BC Turna HZ. Pembrolizumab versus chemotherapy for previously untreated, PD-L1-expressing, locally advanced or metastatic non-small-cell lung cancer (KEYNOTE-042): a randomised, open-label, controlled, phase 3 trial. Lancet (2019) 393(10183):1819–30. 10.1016/S0140-6736(18)32409-7 30955977

[4] Rittmeyer A Barlesi F Waterkamp D Park K Ciardiello F von Pawel J. Atezolizumab versus docetaxel in patients with previously treated non-small-cell lung cancer (OAK): a phase 3, open-label, multicentre randomised controlled trial. Lancet (2017) 389(10066):255–65. 10.1016/S0140-6736(16)32517-X PMC6886121 27979383

[5] Shen X Zhao B. Efficacy of PD-1 or PD-L1 inhibitors and PD-L1 expression status in cancer: meta-analysis. BMJ (2018) 362:k3529. 10.1136/bmj.k3529 30201790 PMC6129950

[6] Ilie M Long-Mira E Bence C Butori C Lassalle S Bouhlel L. Comparative study of the PD-L1 status between surgically resected specimens and matched biopsies of NSCLC patients reveal major discordances: a potential issue for anti-PD-L1 therapeutic strategies. Ann Oncol (2016) 27(1):147–53. 10.1093/annonc/mdv489 26483045

[7] Nath A Neyaz Z Hashim Z Agrawal V Richa M. Role of Percutaneous Computed Tomography-guided Lung Biopsy in Non-resolving Consolidation and Identification of Clinical and High-resolution Computed Tomography Characteristics Predicting Outcome. J Clin Imaging Sci (2019) 9:48. 10.25259/JCIS_126_2019 31819825 PMC6884987

[8] Chang YY Chen CK Yeh YC Wu MH. Diagnostic feasibility and safety of CT-guided core biopsy for lung nodules less than or equal to 8 mm: A single-institution experience. Eur Radiol (2018) 28(2):796–806. 10.1007/s00330-017-5027-1 28884222

[9] Guo W Hao B Chen HJ Zhao L Luo ZM Wu H. PET/CT-guided percutaneous biopsy of FDG-avid metastatic bone lesions in patients with advanced lung cancer: a safe and effective technique. Eur J Nucl Med Mol Imaging (2017) 44(1):25–32. 10.1007/s00259-016-3455-9 27444682 PMC5121178

[10] Fei B Schuster DM. PET Molecular Imaging-Directed Biopsy: A Review. AJR Am J Roentgenol (2017) 209(2):255–69. 10.2214/AJR.17.18047 PMC5669368 28504563

[11] Intepe YS Metin B Sahin S Kaya B Okur A. Our transthoracic biopsy practices accompanied by the imaging process: The contribution of positron emission tomography usage to accurate diagnosis. Acta Clin Belg (2016) 71(4):214–20. 10.1080/17843286.2016.1155810 27142092

[12] Klaeser B Mueller MD Schmid RA Guevara C Krause T Wiskirchen J. PET-CT-guided interventions in the management of FDG-positive lesions in patients suffering from solid malignancies: initial experiences. Eur Radiol (2009) 19(7):1780–5. 10.1007/s00330-009-1338-1 19238391

[13] Hao B Zhao L Luo NN Ruan D Pang YZ Guo W. Is it sufficient to evaluate bone marrow involvement in newly diagnosed lymphomas using (18)F-FDG PET/CT and/or routine iliac crest biopsy? A new approach of PET/CT-guided targeted bone marrow biopsy. BMC Cancer (2018) 18(1):1192. 10.1186/s12885-018-5104-0 30497426 PMC6267895

[14] Guo W Hao B Luo N Ruan D Guo X Chen HJ. Early re-staging and molecular subtype shift surveillance of locally recurrent or metastatic breast cancer: A new PET/CT integrated precise algorithm. Cancer Lett (2018) 418:221–9. 10.1016/j.canlet.2018.01.019 29337111

[15] Zhao L Zhuang Y Fu K Chen P Wang Y Zhuo J. Usefulness of [(18)F]fluorodeoxyglucose PET/CT for evaluating the PD-L1 status in nasopharyngeal carcinoma. Eur J Nucl Med Mol Imaging (2020) 47(5):1065–74. 10.1007/s00259-019-04654-4 31897588

[16] Williams GH Nicholson AG Snead DRJ Thunnissen E Lantuejoul S Cane P. Inter-observer Reliability of Programmed Cell Death Ligand-1 Scoring Using the VENTANA PD-L1 (SP263) Assay in Non-Small Cell Lung Cancer. J Thorac Oncol (2019) 15(4):550–5. 10.1016/j.jtho.2019.11.010 31778799

[17] Rebelatto MC Midha A Mistry A Sabalos C Schechter N Li X. Development of a programmed cell death ligand-1 immunohistochemical assay validated for analysis of non-small cell lung cancer and head and neck squamous cell carcinoma. Diagn Pathol (2016) 11(1):95. 10.1186/s13000-016-0545-8 27717372 PMC5055695

[18] Landis JR Koch GG. The measurement of observer agreement for categorical data. Biometrics (1977) 33(1):159–74. 843571

[19] Li C Huang C Mok TS Zhuang W Xu H Miao Q. Comparison of 22C3 PD-L1 Expression between Surgically Resected Specimens and Paired Tissue Microarrays in Non-Small Cell Lung Cancer. J Thorac Oncol (2017) 12(10):1536–43. 10.1016/j.jtho.2017.07.015 28751245

[20] Topalian SL Hodi FS Brahmer JR Gettinger SN Smith DC McDermott DF. Five-Year Survival and Correlates Among Patients With Advanced Melanoma, Renal Cell Carcinoma, or Non-Small Cell Lung Cancer Treated With Nivolumab. JAMA Oncol (2019) 5(10):1411–20. 10.1001/jamaoncol.2019.2187 PMC6659167 31343665

[21] Garon EB Hellmann MD Rizvi NA Carcereny E Leighl NB Ahn MJ. Five-Year Overall Survival for Patients With Advanced NonSmall-Cell Lung Cancer Treated With Pembrolizumab: Results From the Phase I KEYNOTE-001 Study. J Clin Oncol (2019) 37(28):2518–27. 10.1200/JCO.19.00934 PMC6768611 31154919

[22] Munari E Zamboni G Lunardi G Marchionni L Marconi M Sommaggio M. PD-L1 Expression Heterogeneity in Non-Small Cell Lung Cancer: Defining Criteria for Harmonization between Biopsy Specimens and Whole Sections. J Thorac Oncol (2018) 13(8):1113–20. 10.1016/j.jtho.2018.04.017 29704674

[23] Lantuejoul S Sound-Tsao M Cooper WA Girard N Hirsch FR Roden AC. PD-L1 Testing for Lung Cancer in 2019: Perspective From the IASLC Pathology Committee. J Thorac Oncol (2019) 15(4):499–519. 10.1016/j.jtho.2019.12.107 31870882

[24] Kitazono S Fujiwara Y Tsuta K Utsumi H Kanda S Horinouchi H. Reliability of Small Biopsy Samples Compared With Resected Specimens for the Determination of Programmed Death-Ligand 1 Expression in Non–Small-Cell Lung Cancer. Clin Lung Cancer (2015) 16(5):385–90. 10.1016/j.cllc.2015.03.008 25937270

[25] Tsunoda A Morikawa K Inoue T Miyazawa T Hoshikawa M Takagi M. A prospective observational study to assess PD-L1 expression in small biopsy samples for non-small-cell lung cancer. BMC Cancer (2019) 19(1):546. 10.1186/s12885-019-5773-3 31174496 PMC6555021

[26] Thunnissen E Kerr KM Dafni U Bubendorf L Finn SP Soltermann A. Programmed death-ligand 1 expression influenced by tissue sample size. Scoring based on tissue microarrays’ and cross-validation with resections, in patients with, stage I-III, non-small cell lung carcinoma of the European Thoracic Oncology Platform Lungscape cohort. Mod Pathol (2019) 33(5):792–801. 10.1038/s41379-019-0383-9 31740722

[27] Levy EB Fiel MI Hamilton SR Kleiner DE McCall SJ Schirmacher P. State of the Art: Toward Improving Outcomes of Lung and Liver Tumor Biopsies in Clinical Trials-A Multidisciplinary Approach. J Clin Oncol (2020) 38(14):1633–40. 10.1200/JCO.19.02322. JCO1902322. PMC7351328 32134701

[28] McLaughlin J Han G Schalper KA Carvajal-Hausdorf D Pelekanou V Rehman J. Quantitative Assessment of the Heterogeneity of PD-L1 Expression in Non-Small-Cell Lung Cancer. JAMA Oncol (2016) 2(1):46–54. 10.1001/jamaoncol.2015.3638 26562159 PMC4941982

[29] Wang Y Zhao N Wu Z Pan N Shen X Liu T. New insight on the correlation of metabolic status on (18)F-FDG PET/CT with immune marker expression in patients with non-small cell lung cancer. Eur J Nucl Med Mol Imaging (2019) 47(5):1127–36. 10.1007/s00259-019-04500-7 31502013

[30] Kasahara N Kaira K Bao P Higuchi T Arisaka Y Erkhem-Ochir B. Correlation of tumor-related immunity with 18F-FDG-PET in pulmonary squamous-cell carcinoma. Lung Cancer (2018) 119:71–7. 10.1016/j.lungcan.2018.03.001 29656756

[31] Kaira K Shimizu K Kitahara S Yajima T Atsumi J Kosaka T. 2-Deoxy-2-[fluorine-18] fluoro-d-glucose uptake on positron emission tomography is associated with programmed death ligand-1 expression in patients with pulmonary adenocarcinoma. Eur J Cancer (2018) 101:181–90. 10.1016/j.ejca.2018.06.022 30077123

[32] Lopci E Toschi L Grizzi F Rahal D Olivari L Castino GF. Correlation of metabolic information on FDG-PET with tissue expression of immune markers in patients with non-small cell lung cancer (NSCLC) who are candidates for upfront surgery. Eur J Nucl Med Mol Imaging (2016) 43(11):1954–61. 10.1007/s00259-016-3425-2 27251642

[33] Evangelista L. The prediction of response to immunotherapy in non-small cell lung cancer patients by 18F-FDG PET/CT. J Thorac Dis (2019) 11(11):E221–E3. 10.21037/jtd.2019.10.19 PMC6940221 31903287

[34] Grizzi F Castello A Lopci E. Is it time to change our vision of tumor metabolism prior to immunotherapy? Eur J Nucl Med Mol Imaging (2018) 45(6):1072–5. 10.1007/s00259-018-3988-1 29532102

[35] Boothman AM Scott M Ratcliffe M Whiteley J Dennis PA Wadsworth C. Impact of Patient Characteristics, Prior Therapy, and Sample Type on Tumor Cell Programmed Cell Death Ligand 1 Expression in Patients with Advanced NSCLC Screened for the ATLANTIC Study. J Thorac Oncol (2019) 14(8):1390–9. 10.1016/j.jtho.2019.04.025 31063864

[36] Ratcliffe MJ Sharpe A Midha A Barker C Scott M Scorer P. Agreement between Programmed Cell Death Ligand-1 Diagnostic Assays across Multiple Protein Expression Cutoffs in Non-Small Cell Lung Cancer. Clin Cancer Res (2017) 23(14):3585–91. 10.1158/1078-0432.CCR-16-2375 28073845

[37] Brunnstrom H Johansson A Westbom-Fremer S Backman M Djureinovic D Patthey A. PD-L1 immunohistochemistry in clinical diagnostics of lung cancer: inter-pathologist variability is higher than assay variability. Mod Pathol (2017) 30(10):1411–21. 10.1038/modpathol.2017.59 28664936

[38] Tsao MS Kerr KM Kockx M Beasley MB Borczuk AC Botling J. PD-L1 Immunohistochemistry Comparability Study in Real-Life Clinical Samples: Results of Blueprint Phase 2 Project. J Thorac Oncol (2018) 13(9):1302–11. 10.1016/j.jtho.2018.05.013 PMC8386299 29800747

[39] Yeh J Marrone KA Forde PM. Neoadjuvant and consolidation immuno-oncology therapy in stage III non-small cell lung cancer. J Thorac Dis (2018) 10(Suppl 3):S451–S9. 10.21037/jtd.2018.01.109 PMC5861269 29593890

[40] Forde PM Chaft JE Smith KN Anagnostou V Cottrell TR Hellmann MD. Neoadjuvant PD-1 Blockade in Resectable Lung Cancer. N Engl J Med (2018) 378(21):1976–86. 10.1056/NEJMoa1716078 PMC6223617 29658848

