# Supplementary material for: Concordance of PD-L1 Status Between Image-Guided Percutaneous Biopsies and Matched Surgical Specimen in Non-Small Cell Lung Cancer
Source: Front Oncol. 2021 Feb 23;10:551367. doi: 10.3389/fonc.2020.551367 (PMC7940543; doi:10.3389/fonc.2020.551367)
Supplement: Supplementary file 1 [file DataSheet_1.docx]

Supplemental Table 1. PD-L1 diagnostic assays approved for immunotherapies and their associated indications in NSCLC

| **Immunotherapy** | **Pembrolizumab** | **Nivolumab** | **Durvalumab** | **Atezolizumab** |
| --- | --- | --- | --- | --- |
| **Antibody clone** | 22C3/SP263 | 28-8/SP263 | SP263 | SP142 |
| **Assay platform** | DAKO/Ventana | DAKO/Ventana | Ventana | Ventana |
| **Thresholds in clinical trials** | Tumor Proportion Score (TPS) ≥1% TPS ≥50% | TPS ≥1%, TPS ≥5% TPS ≥10% | TPS ≥1%, TPS ≥25% | Tumor cells (TC) ≥50% (TC3) and immune cells (IC) ≥10% (IC3)^a^ |
| **Thresholds in therapy** | TPS ≥1% first-line TPS ≥50% first-line | TPS ≥1% second-line | Not applicable (NA) | NA |
| **Diagnostic status** | Companion | Complementary | Complementary | Complementary |

^a^TC0, <1%; TC1, 1%–5%; TC2, 5%–50%; TC3, ≥50%; IC0, <1%; IC1, 1%–5%; IC2, 5%–10%; IC3, ≥10%

Notes: Companion, testing required; Complementary: testing not required

Supplemental Table 2. PD-L1 expression of surgical resected specimen and SUVmax in the PET/CT group

| **PD-L1 expression** | **Score*** | **SUVmax** |
| --- | --- | --- |
| 0% | 0 | 7.5 |
| 10% | 1 | 1.0 |
| 1% | 1 | 3.1 |
| 0% | 0 | 1.2 |
| 0% | 0 | 1.0 |
| 0% | 0 | 1.5 |
| 0% | 0 | 0.9 |
| 25% | 1 | 1.5 |
| 20% | 1 | 5.7 |
| 0% | 0 | 7.3 |
| 15% | 1 | 8.1 |
| 0% | 0 | 0.8 |
| 0% | 0 | 1.8 |
| 0% | 0 | 1.8 |
| 55% | 2 | 1.9 |
| 0% | 0 | 3.0 |
| 0% | 0 | 2.8 |
| 25% | 1 | 5.1 |
| 0% | 0 | 8.9 |
| 0% | 0 | 1.6 |
| 40% | 1 | 6.0 |
| 0% | 0 | 4.9 |
| 5% | 1 | 1.3 |
| 50% | 2 | 1.5 |
| 6% | 1 | 3.6 |
| 0% | 0 | 2.7 |
| 0% | 0 | 6.6 |
| 1% | 1 | 3.9 |
| 40% | 1 | 7.3 |
| 0% | 0 | 3.9 |
| 25% | 1 | 7.1 |
| 20% | 1 | 1.5 |
| 0% | 0 | 1.9 |
| 30% | 1 | 6.7 |
| 1% | 1 | 6.6 |
| 0% | 0 | 11.9 |
| 100% | 2 | 11.7 |
| 80% | 2 | 10.1 |
| 25% | 1 | 7.6 |
| 0% | 0 | 5.3 |
| 10% | 1 | 10.9 |
| 75% | 2 | 16.6 |
| 0% | 0 | 8.9 |
| 30% | 1 | 2.9 |
| 0% | 0 | 3.2 |
| 10% | 1 | 2.2 |
| 0% | 0 | 8.9 |
| 10% | 1 | 3.0 |
| 10% | 1 | 4.8 |
| 10% | 1 | 3.1 |
| 0% | 0 | 2.4 |
| 0% | 0 | 13.1 |
| 0% | 0 | 6.1 |
| 100% | 2 | 7.4 |
| 0% | 0 | 1.8 |
| 25% | 1 | 8.7 |
| 1% | 1 | 7.2 |
| 0% | 0 | 1.9 |
| 0% | 0 | 14.8 |
| 65% | 2 | 8.4 |
| 0% | 0 | 7.4 |
| 80% | 2 | 13.7 |
| 1% | 1 | 10.9 |
| 3% | 1 | 6.0 |
| 0% | 0 | 6.4 |
| 0% | 0 | 4.6 |
| 0% | 0 | 0.9 |
| 30% | 1 | 9.4 |
| 2% | 1 | 1.5 |
| 40% | 1 | 4.0 |

*Scoring rule: <1%, 0; 1%-50%, 1; ≥50%, 2.

Supplemental Table 3. Comparison of clinicodemographic characteristics in the CT and the PET/CT groups

| **Characteristic** | **CT-guided biopsy group** | **PET/CT-guided biopsy group** | **P value** |
| --- | --- | --- | --- |
| Age (years) |  |  | 0.71 |
| <65 | 40 | 39 |  |
| ≥65 | 28 | 31 |  |
| Sex |  |  | 0.86 |
| Male | 36 | 36 |  |
| Female | 32 | 34 |  |
| Smoking history |  |  | 0.76 |
| Nonsmoker | 41 | 44 |  |
| Smoker | 27 | 26 |  |
| Histology |  |  | 0.39 |
| Squamous cell carcinoma | 9 | 13 |  |
| Adenocarcinoma | 59 | 57 |  |
| Clinical stage (International Association for the Study of Lung Cancer 8th) |  |  | 0.08 |
| I | 43 | 34 |  |
| II/III | 25 | 36 |  |
| Derived neutrophil–lymphocyte ratio |  |  | 0.67 |
| <3 | 66 | 67 |  |
| ≥3 | 2 | 3 |  |
| Lactate dehydrogenase (U/L) |  |  | 1 |
| <240 | 64 | 65 |  |
| ≥240 | 4 | 5 |  |
| Body mass index |  |  | 0.63 |
| <25 | 49 | 53 |  |
| ≥25 | 19 | 17 |  |
| Tumor Proportion Score |  |  | 0.47 |
| <1% | 25 | 33 |  |
| 1%–49% | 34 | 29 |  |
| ≥50% | 9 | 8 |  |
| SUVmax^a^ |  |  |  |
| Median (range) |  | 4.9 (0.8–16.6) |  |

Note: ^a^Data include patients who were in the PET/CT group.
